# What is the evidence that a pharmacy team working in an acute or emergency medicine department improves outcomes for patients: A systematic review

**DOI:** 10.1002/prp2.1007

**Published:** 2022-09-14

**Authors:** Ekta Punj, Abbie Collins, Nirlep Agravedi, John Marriott, Elizabeth Sapey

**Affiliations:** ^1^ Clinical Research Network University of Birmingham Birmingham UK; ^2^ Pharmacy Department University Hospitals Birmingham NHS Foundation Trust Birmingham UK; ^3^ University of Birmingham Birmingham UK; ^4^ PIONEER, HDRUK Health Data Hub in Acute Care Birmingham UK; ^5^ Institute of Inflammation and Ageing University of Birmingham Birmingham UK; ^6^ Acute Medicine University Hospitals Birmingham NHS Foundation Trust Birmingham UK

**Keywords:** emergency medicine, medication errors, medication reconciliation, pharmac*

## Abstract

Pharmacy services within hospitals are changing, with more taking on medication reconciliation activities. This systematic review was conducted to determine the measured impacts of Pharmacy teams working in an acute or emergency medicine department. The protocol followed the Preferred Reporting Items for Systematic Reviews and Meta‐Analyses (PRISMA) guidelines and was prospectively registered on PROSPERO, National Institute for Health and Care Research, UK registration number: CRD42020187487. The systematic review had two co‐primary aims: a reduction in the number of incorrect prescriptions on admission by comparing the medication list from primary care to secondary care, and a reduction in the severity of harm caused by these incorrect prescriptions; chosen to determine the impact of pharmacy‐led medication reconciliation services in the emergency and acute medicine setting. Seventeen articles were included. Fifteen were non‐randomized controlled trials and two were randomized controlled trials. The number of patients combined for all studies was 7630. No studies included were based within the UK. All studies showed benefits in terms of a reduction in medicine errors and patient harm, compared to control arms. Nine articles were included in a statistical analysis comparing the pharmacy intervention arm with the non‐pharmacy control arm, with a Chi^2^ of 101.10 and *I* ^2^ value = 92%. However, studies were heterogenous with different outcome measures and many showed evidence of bias. The included studies consistently indicated that pharmacy services based within acute or emergency medicine departments in hospitals were associated with fewer medication errors. Further studies are needed to understand the health and economic impact of deploying a pharmacy service in acute medical settings including out‐of‐hours working.

## Graphical abstract

Attached is the graphical abstract summarising our article's methods and outcomes.

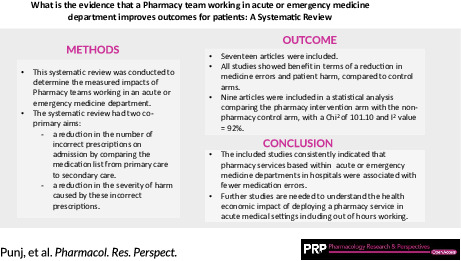

## 1 INTRODUCTION

Medication use is almost ubiquitous in the prevention, treatment, and management of disease.^1^ NHS Digital identified that in England the 2019–2020 medication spend was listed as £20.9 billion with around £11.7 billion being spent on the medication used in hospitals.^2^ Therapy options have become more complex across all disease areas and multi‐morbidity is increasing in our aging population. This has resulted in high levels of polypharmacy (defined as the concurrent use of multiple medication items by one individual^3^). The 2021 Ridge report described that 15% of people in England are taking more than five medications a day, with 7% on more than eight medications a day.^4^

Medication errors have been defined as patient safety incidents where an error has occurred in the process of prescribing, administering, monitoring, or providing advice on medication. They can be divided into two categories: errors of commission or errors of omission.^5^ The former includes the wrong medicine or dose being given. The latter is when a dose or medication is missed or monitoring is not implemented.^6^ Research reviewed by NHS England has suggested an error rate of 7% within a hospital setting and 5% within general practice,^6^ indicating that medication errors are common.

Adverse drug reactions (ADRs) are defined as appreciably harmful or unpleasant reactions resulting from an intervention related to the use of a medication product^7^ and problematic polypharmacy is defined as the inappropriate prescribing of multiple medication.^3^ These are thought to occur in 10%–20% of in‐patient hospital admissions^5^ and negatively impact patient care, increasing hospital length of stay and overall NHS costs.^4^ ADRs are more common in the presence of problematic polypharmacy.

The transfer of care between care providers is an area of high risk for medication errors. It had been estimated that 30%–70% of patients had an error or unintentional change to their medication during transitions from acute services.^8^ The reasons for these errors are complex but may reflect current limitations within data sharing across prescribing systems.^4^ Also, transitions of care are most often made during an emergency or unplanned healthcare event (such as the transition from primary care or care home to hospital emergency departments). These tend to be busy areas of clinical service, with patients being seen by multiple health staff, who are caring for many patients at the same time. There were 25 million Emergency Department attendances in 2019/20 with an increase of 24% between 2011/12 and 2020/21.^9^

It is unlikely that the increasing demand for acute services and acute transitions of care will abate, and new strategies are needed to reduce medication errors, including more effective use of the clinical multi‐disciplinary team.

Medication reconciliation is the process of identifying an accurate list of a person's current medications and comparing them with the current list in use.^1^ This includes any treatments supplied through additional healthcare providers and over‐the‐counter or complementary medication. Once discrepancies are identified, they should be resolved promptly. The National Institute for Health and Care Excellence (NICE), advises that medicine reconciliation should be completed within 24 h or sooner if clinically necessary when a person moves from one care setting to another.^1^

A number of systematic reviews and meta‐analyses have identified the benefits of pharmacy‐led medication review reducing but not eradicating medicine errors within the emergency department,^10^ transitions of care between secondary and primary care,^11^, ^12^ and the low‐level impact on hospital readmissions.^13^ However, of these few have been conducted in both an acute/emergency setting, and none have specifically assessed the impact of a pharmacy team aiding with medication histories and reconciliation during acute medical admissions. This SR was conducted to determine the outcome of a Pharmacy team working in an acute or emergency medicine department.

## 2 METHOD

### 2.1 Protocol and registration

The systematic review protocol was made using the Preferred Reporting Items for Systematic Reviews and Meta‐Analyses (PRISMA, UK) guideline^14^ and the Cochrane Handbook guidance, UK.^15^ It was registered on PROSPERO, NIHR, UK (registration number: CRD42020187487—see https://www.crd.york.ac.uk/prospero/display_record.php?RecordID=187487).

### 2.2 Eligibility criteria

#### 2.2.1 Study design

Studies were selected on the inclusion and exclusion criteria described in Table 1.

**TABLE 1:** Inclusion and exclusion criteria for the systematic review

Inclusion	Exclusion:
Set within an acute or emergency medicine department	Any studies not assessing a pharmacy service
The intervention consisted of a pharmacy service (being provided by pharmacists, clinical pharmacy technicians, pre‐registration pharmacists, or pharmacy students)	Those which had no clear non‐pharmacy control for the medication history or reconciliation intervention to be measured parallel to
There was no restriction by medical or surgical condition	Outcomes not associated with medication discrepancies
The pharmacy service consisted of a full medication history or reconciliation, in comparison to the control of having no pharmacy service	Studies not set within an acute medicine or an emergency medicine hospital department
There were no restrictions on age	Case studies were excluded, and any case series with less than 10 participants
This was inclusive of: observational studies randomized studies case series with 10 participants or more all ages (including adult and pediatric studies) all languages all publication dates all available countries no minimum duration of follow up	Studies using other services or whose focus was solely reviewing the impact of medication‐taking tools were excluded during this review
	Previous systematic reviews and meta‐analyses were excluded in the full data analysis. (The findings of existing systematic reviews and meta‐analyses were discussed within the discussion section. Primary studies from these reviews were searched and included if these met the inclusion criteria.)

*Note*: Criteria are also available on PROSPERO (registration number: CRD42020187487 – see https://www.crd.york.ac.uk/prospero/display_record.php?RecordID=187487).

### 2.3 Outcomes

The co‐primary outcomes were.

1. A reduction in medication errors, defined as errors in medication reconciliation; errors in medication prescribing (dose, formulation, frequency, type of administration); errors in medication transcription (inclusive of commissions (unintentional new medications) or omissions (unintentionally missed medications)); medication interactions; a failure of appropriate dose adjustment for patient characteristics (age, weight, organ function).
2. A reduction in patient harm, defined as a statistically significant risk reduction value.

If data were available, a subgroup analysis was planned to assess the impact of the interventions on the speed of processing patients through the acute healthcare service, whether staff experience (seniority grade) affected the number of errors identified and whether polypharmacy and multimorbidity affected medication error rates.

### 2.4 Search strategy

#### 2.4.1 Information sources

Health databases were searched using index/MeSH (Medical Subject Headings) and strings of keyword terms, which were pharmac*, medication reconciliation, and emergency medicine. Searches were undertaken with no language or age restrictions applied. Databases included the Cochrane Database of Systematic Reviews, MEDLINE (via Ovid), Embase (via Ovid), CINAHL Plus (via EBSCO), MEDLINE in Process (via Ovid), Cochrane Central Register of Controlled Trials (CENTRAL), PsycInfo (via Ovid), Healthcare Management Information Consortium database and Web of Science. Each database library was searched using terminology specific to the database. This is detailed in the online supplement including the dates of searches. ClinicalTrials.gov and the International Clinical Trials Registry Platform were searched. Where possible, non‐English articles were translated. Each of the included publications had their reference lists hand searched for all included studies, relevant systematic reviews, and primary studies. Where needed, authors of the relevant studies and reviews were contacted to clarify published and unpublished data: this was completed up to a maximum of three times before being abandoned. A forward citation search was completed using Web of Science. Articles obtained through the databases were exported into Rayyan® Qatar Foundation, Qatar^16^ to allow duplicates to be identified and removed.

#### 2.4.2 Study selection

Each paper was independently reviewed by at least two authors (EP and AC/NA), and disagreements were resolved by discussions with a third reviewer. Reasons for inclusion or exclusion were documented.

After the initial screening, articles were exported into EndNote X9® (Clarivate) for full‐text screening. Results were then exported to Microsoft Excel® (Microsoft) for review. Two authors (EP and AC/ NA) read through the full articles using the predefined inclusion and exclusion criteria, disagreements were resolved via a discussion with a third reviewer, and reasons for outcomes were noted.

#### 2.4.3 Data extraction

A standardized form for data synthesis was built and piloted before use. The data were synthesized using this standardized form by the first author (EP). Duplicate records were removed and the reason for exclusion was recorded. The articles were divided and checked between two authors (AC/NA) to check for inaccuracies. Any disagreements with missing data were resolved by discussion with a third reviewer.

#### 2.4.4 Risk of bias and quality assessment

The risk of bias was assessed using two tools. Randomized controlled trials (RCT) were assessed using the Cochrane Risk of bias tool (ROB2® Cochrane).^17^ For non‐randomized controlled trials (NRCTs), the Risk Of Bias In Non‐randomized‐Studies– of Interventions (ROBINS‐I®, Cochrane)^18^ was used. The risk of bias was assessed in seven domains. Outcomes were presented using Risk‐Of‐Bias Visualization (ROBVIS®, Cochrane).^19^

Each tool was applied by one author (AC/NA) and checked by a second author (EP). Any queries with missing data were resolved by discussion by a third author.

#### 2.4.5 Data synthesis

RevMan® (Cochrane, UK)^20^ was used to combine the data and perform statistical analysis. Heterogeneity between studies was reviewed using a visual inspection of the forest plot: Chi^2^ test and I^2^ tests were also completed. Where data were limited, descriptive analyses were used.

## 3 RESULTS

**FIGURE 1:**
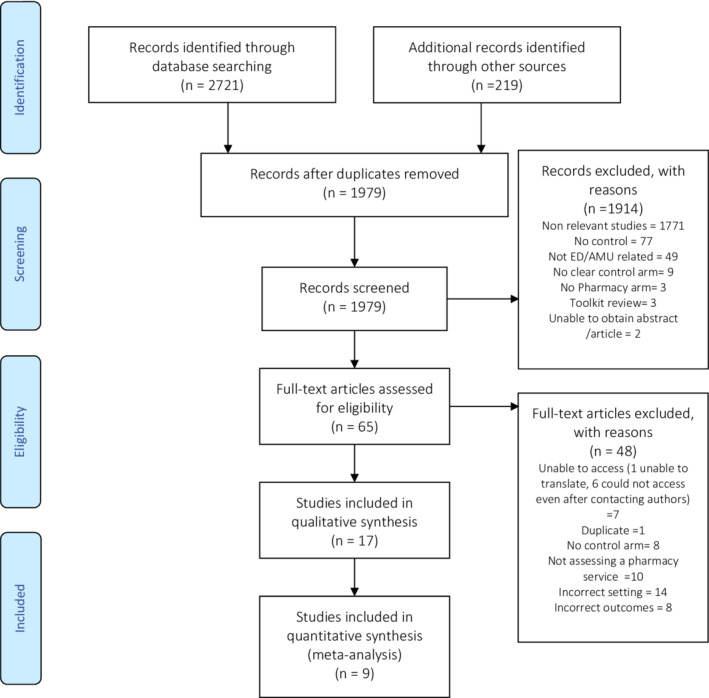
The article review process with reasons for any article exclusions. Preferred Reporting Items for Systematic Reviews and Meta‐Analyses® (PRISMA) UK^14^ diagram for the review, included in the online supplement.

Of the 2110 eligible citations (once duplicates were removed), 65 met the inclusion criteria, as shown in the PRISMA® UK flow diagram (Figure 1). When full‐text articles were assessed, a further 48 were excluded, leaving 17 articles for inclusion.

Of the 17 articles that were included in the qualitative synthesis, nine articles were used in the subgroup statistical analysis, owing to a high level of heterogeneity across the 17 articles.

Of the included studies, 15 were non‐randomized controlled trials (NRCTs)^21^, ^22^, ^23^, ^24^, ^25^, ^26^, ^27^, ^28^, ^29^, ^30^, ^31^, ^32^, ^33^, ^34^, ^35^ and two were randomized controlled trials (RCT),^36^, ^37^ all of which were available in English. The number of patients combined for all studies was 7630. The statistical review included 3001 patients. No studies included were based within the UK. 5457 (71.5%) participants were from four studies^25^, ^26^, ^28^, ^30^ within Europe. Two thousand one hundred and seventy‐three (28.5%) participants were from 13 studies^11^, ^21^, ^22^, ^23^, ^24^, ^27^, ^29^, ^31^, ^33^, ^34^, ^35^, ^36^, ^37^ outside of Europe. Table 2 provides an overview of all studies within the review.

**TABLE 2:** The studies and main results included within this review

Author	Study background	Design	Patient allocation	Inclusion	Exclusion	Control (C) and intervention (I)	Outcomes overall	Results control (C) and Intervention (I)
Crook M, et al. 2007^21^	Australia, single site ED, May 6th ‐ July 6th, 2005 (6 weeks)	Post medical history review	100 participants (same group in the intervention and control arm)	Patients within the Emergency Department, over the age of 70 years old, taking five or more regular medications, had three or more clinical comorbidities, **or** had been discharged from the hospital three months prior to the study	Under the age of 70 years old. Not wishing to participate. Unable to communicate due to language difficulties when an interpreter could not be employed. Unable to give consent (reasons included: severe dementia, deaf, too ill, and psychosocial issues). Extra precautions required (e.g., multi‐resistant Staphylococcus aureus infection)	(C): ED doctor. (I): pharmacy researcher (pharmacy honors student)	Accuracy of medication histories, recording of ADRs, time taken to complete a medication history, medication‐related problems, and pharmacist interventions	**Medication errors during reconciliation** ‐ 1152 medications recorded (I) versus 189 (C). ‐ 966 (83.9%) medication errors. ‐ 563 (48.9%) omission of medication. ‐ 869.4 (90%) omission of either dose or frequency of medication. ‐ The mean number of discrepancies per patient was 9.7 (standard deviation [SD] =4.7). ‐ Medication‐related problem rate was 0.55 per patient. **Dose adjustments for patient characteristics on admission** ‐ 29 events.
Hayes BD, et al. 2007^22^	USA, single site ED, March 2006–April 2006 (8 weeks)	Pilot study Retrospective chart review and prospective intervention	160 participants: 100 in the control. 60 participants in the intervention.	Patients admitted through ED. 18 years old or over, 9 am‐5 pm Mon‐Fri, in March 2006 (C) and in April 2006 (I).	No specific exclusion criteria, but ended up excluding patients with intubation, excessive trauma, or other medical conditions limiting communication were excluded during the study	(C): admitting physician. (I): pharmacist	Medication reconciliation form compliance. Medication discrepancies (accuracy) and allergy documentation	**Medication errors during reconciliation** ‐ 117 errors out of the 601 medications recorded (C) versus 2 errors out of the 378 medications recorded (I). ‐ The mean SD number of errors per form 1.7 +/− 2.1 (C) versus 0.3+/− 0.7 (I) (p = 0.001). ‐ Percentage of forms with 1 error was significantly higher in the control group 59% (C) versus 3% (I) (p = 0.001). ‐59% of medication histories (C) contained at least one error that required follow‐up by a pharmacist. ‐71% of 117 errors to be due to omissions.
Kent AJ, et al. 2009^23^	Canada, single site ED, September 25th – November 17th, 2006 (8 weeks).	Retrospective analysis of medical records post nurse /physician medication history and reconciliation.	200 participants: Analysis completed on: 86 in the baseline control. 86 in the post‐intervention.	A convenience sample taken from a randomly generated list of patients admitted through the ED 8 a.m.–4 p.m.	Those unable to communicate and who did not have a caregiver available. Patients who discharged or who passed away, within 24 h of admission were excluded.	(C): nurses / physicians. (I): pharmacist.	Medication discrepancies.	**Medication errors during reconciliation** ‐124 medication discrepancies in 98 patients. ‐519 (C) and 543 (I) medications at home. ‐170 errors per 100 admissions (C), 80 errors per 100 admissions (I) (53% reduction). ‐Number of medication discrepancies per medication record declined from 1.7 (C) to 0.8 (I). ‐Number of medication records with at least 1 discrepancy was 59 (C) and 39 (I) (34% reduction). ‐82 (66%) omissions.
Vasileff HM, et al. 2009^24^	Australia, single site, ED, April – July 2007	Usual practice arm (6 weeks) compared with pharmacist charting arm (5 weeks)	74 participants: 45 in the control. 29 in the intervention	Convenience sample within the hours of 08:00 and 17:00 in ED. Patients aged 60 years or older, who took four or more regular medications and had three or more clinical co‐morbidities and/or had at least one previous hospital admission within the past 3 months	If they were unable to communicate due to language difficulties, under psychiatric care, and/or were unable to give consent	(C): doctor. (I): pharmacy researcher	Frequency and severity of medication discrepancies and errors	**Errors in medication reconciliation** *−*111, patients with 1 or more error: 75.6% (C) versus 3.3% (I) unintentional discrepancies. Average number per patient +/− SD 2.51 +/− 2.37 (C), 0.034 +/− 0.19 (I). −57% omissions. **Reduction in patient harm** *From the medication reconciliation discrepancies in the control arm*: 6% (7/111 errors in total) very significant impact. 52% (58/111 errors in total) significant impact. 40% (44/111 errors in total) minor impact. 2% (2/111 errors in total) no impact
De Winter S, et al. 2010^25^	Belgium, single site, ED, February 2007–August 2008, (19 months)	Pharmacists obtained histories for admitted patients separate to the physicians	3594 participants (same group in the intervention and control arm)	08:30–17:00 during the week, depending on the availability of pharmacy staff within ED	If they were younger than 16 years old. Intubated/mechanically ventilated, poisoned, and psychiatric patients were excluded.	(C): physician. (I): a clinical pharmacist /a well‐trained pharmacy technician	Medication discrepancies	**Medication errors during reconciliation** −2134 (59% CI+/−0.8%) (C) medication histories differed from the (I). −5963 discrepancies found within them. ‐Omission of medication 61% (95% CI 60.4 to 61.6%) and omission of dose 1089 (18%, 95% CI 17.6% to 18.4%). **Errors in new medication prescribing** −388 (6.5% +/− 0.3%) commission of medication
Becerra‐Camargo J, et al. 2013^36^	Columbia, multisite, ED, October 26th ‐ November 30th, 2012	Double‐blind, randomized, controlled, parallel‐group study	270 participants, analysis completed on: Control 125. Intervention 117	Enrolled 24 h a day if they met the inclusion criteria. Aged 18 years or older. Admitted through ED taking at least one prescription medication or prescribed a minimum of one prescription medication before admission, who had been assessed as triage one or two on admission and hospitalized for at least 24 h were eligible for inclusion	Excluded if scheduled for discharge on the same day, unable to answer the questions needed to complete the study, were unable to communicate due to language difficulties, were under psychiatric care, had a medical record of dementia or confusion, **and/or** were unable to give their consent	(C): doctor. (I): pharmacist	Medication discrepancies. ADRs	**Medication errors during reconciliation** −117 (93.6%) (C) versus 71 (60.7%) (I) had at least one medication discrepancy. The intervention reduced the discrepancy by 33% (*p* < .0001;0.1055), Odds Ratio (OR), 0.05–0.24 95% Confidence Interval (CI). ‐The overall discrepancy rate was 3.35 per patient (SD 3.32). It was 4.23 (SD 3.26) (C) and 2.43 (SD 3.14) (I). −55.1% omissions 66.3% (C) versus 34.3% (I). **Reduction in patient harm.** *From the medication reconciliation discrepancies combined in both arms combined*: −33.4% (271/811 errors in total) discrepancies unlikely to cause potential discomfort or clinical deterioration. −42.7% (346/811 errors in total) discrepancies which could cause moderate discomfort or clinical deterioration. −23.9% (194/811 errors in total) discrepancies potentially resulting in severe discomfort or clinical deterioration. *Errors as a percentage of the total charted prescriptions*: −45.2% (528/1169 errors out of the total recorded prescriptions) control. ‐ 24.2% (283/1169 errors out of the total recorded prescriptions) intervention
van den Bemt PMLA, et al. 2013^26^	The Netherlands, multisite, ED/Acute Admission, 2–4 months over March 2019 –July 2012	Observational study with a pre‐post design	1543 participants (350 mixed‐model hospitals): Control 436 (81 mixed‐model hospitals). Intervention 1107 (269 mixed‐model hospitals)	Patients aged 65 years and older with an acute hospital admission through the ED	Individuals without medications were excluded	(C): physician /nurses. (I): nine hospitals had pharmacy technician and in three hospitals a mixed model consisted of physicians or pharmacy technicians	Medication discrepancies	**Medication errors during reconciliation** ‐One or more unintentional medication discrepancies were reduced from 62% (225/436 (C)) to 32% (183/1107 (I)) OR = 0.16, 95% CI = 0.12–0.21 after the intervention. −438 omissions in 3618 (C) versus 503 omissions in 9277 (I)
de Andres‐Lazaro AM, et al. 2015^28^	Spain, single site, ED, November 2011–March 2012 (4 months).	Prospective interventional study	227 participants: Analysis completed on 214 participants (same group in the intervention and control arm)	Patients recruited at 8 am Monday–Friday. Adults aged over 18 years old	Patients with language barriers or physical status (e.g., disorientated, or sedated patient) were excluded. Also, if the detected discrepancy could not be verified by the physician in charge, they were excluded from the reconciliation error analysis	(C): doctor. (I): pharmacist	Medication discrepancies	**Medication errors during reconciliation** −1596 medications confirmed which had 980 discrepancies. ‐The average discrepancy per patient was 4.58 (4.03 SD). 39% of prescriptions recorded in medication history were correct. −384 omissions of medication and 324 omissions of dose and/or frequency. **Errors in new medication prescribing** −135 medication commissions
Hart C, et al. 2015^29^	Florida, USA, single site, ED, November 2011–February 2012.	Pre–poststudy.	300 participants: Control 150. Intervention 150.	Patients enrolled 7 days a week 1 pm‐9:30 pm. Older than 18 years old, were admitted to the hospital directly from the ED, took at least 3 medications on arrival.	If incapable of providing a medication history.	(C): nurses. (I): pharmacy technicians.	Accuracy of medication histories and types of discrepancies in each group. ADRs and allergy documentation.	**Medication errors during reconciliation** ‐Medication history accuracy 88% (I) and 57% (C) (*p* < .0001). −19 (1.1%) (I) errors compared with 117 (8.3%) (C) Relative Risk (RR) 7.5; *p* < .0001, difference = −0.07 (7%), 95% CI ‐0.086 to −0.055 *p* < .0001. ‐Medication omission (10 versus 59, *p* < .001). **Errors in new medication prescribing** Medication commission 21% in total, 5 (I) versus 23 (C), p = 0.004.
Cater SW, et al. 2015^27^	North Carolina, USA single site, ED, June 1st‐ August 1st, 2008	Prospective cohort study	188 participants: Control 75. Intervention 113	Adults aged 17 and over in ED	Excluded institutionalized (i.e., living in a nursing home, group home, or psychiatric facility), medically unstable, mentally incapacitated without a guardian present, non‐English speaking, or foreign citizens, or those suspected to be under the influence of medications or alcohol	(C): physician. (I): pharmacy technicians	Medication discrepancies	**Medication errors during reconciliation** ‐ 42.2% (661/1566 errors in total) (C). ‐ 57.7% (905/1566 errors in total) (I). −352 (62%) out of 566 (I) errors versus 228 (56%) out of 406 (C) were deemed unjustified. Not statistically significant P = 0.0586 ‐The rate of unjustified changes per patient was 3.14 [SD 2.98] (I) and 3.17 [SD 2.81] (C) p = 0.9570, not significant. −1566 justified and unjustified changes. ‐Total omissions 814 (52%). 483 (53.4%) (I) and 331 (50.1%) (C). **Errors in new medication prescribing** ‐Medication commission 339 (37.5%) (I) and 255 (38.6%) (C). **Reduction in patient harm.** *From the medication reconciliation discrepancies in the control and intervention arm separately*: −35.7% (126/353 errors in total in the intervention group) and 34.7% (79/228 errors in total in the control group) insignificant errors. −34.0% (120/353 errors in total in the intervention group) and 38.6% (88/228 errors in total in the control group) minimal errors. −30.3% (107/353 errors in total in the intervention group) and 26.8% (61/228 errors in total in the control group) insignificant errors. *Errors as a percentage of the total charted prescriptions* −44.9% (661/1473 errors out of the total recorded prescriptions) control. −61.4% (905/1473 errors out of the total recorded prescriptions) intervention
Henriksen JP, et al. 2015^30^	Denmark, single site, ED, March–May 2012, and November 2012‐ January 2013	Pharmacists obtained histories for admitted patients separate to the physicians	113 participants: Analysis completed on 106 participants (same group in the intervention and control arm)	Patients with 3 medications as a minimum at the time of the hospital admission via ED		(C): physician. (I): pharmacy technicians	Medication discrepancies	**Medication errors during reconciliation** −1075 medications recorded. 287 (C) discrepancies (27% of the total prescriptions) and 28 (2% of the total prescriptions) (I). ‐On average there were three discrepancies (C) and less than one (I)
Khalil V, et al. 2016^31^	Australia, single site, Acute Assessment and Admission Unit, August–September 2015 (6‐week period)	Prospective parallel study	110 participants: Control 54. Intervention 56.	All consecutive adult medical patients admitted to the Acute Assessment and Admission unit were included during the h of 8:30 am to 5 pm	Not admitted to the Acute Assessment and Admission unit within 24 h or if they did not have any medications prior to admission or were not a general medical patient	(C): physician. (I): pharmacist	Medication and ADR discrepancies.	**Medication errors during reconciliation** ‐ 43% (238/554 errors out of the recorded prescriptions total) control. ‐ 4.9% (29/595 errors out of the recorded prescriptions total) intervention. −4.41 average error rate per patient in the control to 0.52 errors (*p* < .0001) (relative reduction of 88% *p* < .0001) and 0.43–0.05 errors per order (relative reduction of 89% *p* < .005). ‐ 2 omissions (I) 116 (C). **Reduction in patient harm.** *From the medication reconciliation discrepancies in the control and intervention arm separately*: −0% (0/595 errors in total in the intervention group) and 17.5% (97/554 errors in total in the control group) extreme and high harm. −1% (6/595 errors in total in the intervention group) and 11.6% (64/554 errors in total in the control group) moderate harm. −3.9% (23/595 errors in total in the intervention group) and 13.9% (77/554 errors in total in the control group) low harm. *Errors as a percentage of the total charted prescriptions* −20.7% (238/1149 errors out of the total recorded prescriptions) control. −2.5% (295/1149 errors out of the total recorded prescriptions) intervention.
Mekonnen AB, et al. 2018^32^	Ethiopia, single center, ED, February and August 2016, 6 months.	Prospective single center, pre–post study.	123 patients: Control: 49. Intervention 74.	Eligible patients enrolled in the ED were adults (aged 18 years or over) that had been hospitalized for at least 24 h and taking at least two home/regular medications on admission. Patients were conveniently enrolled on weekdays.		(C): physician. (I): pharmacist.	Medication discrepancies and severity.	**Medication errors during reconciliation** *‐ Patients with at least one unintended discrepancy was reduced from 59% (29/49 (C)) to 10.5% (8/76 (I)) (p < .001)*. *Errors as a percentage of medication errors within each arm*. − 42% (73/174 errors out of the recorded prescriptions total) control ‐ 3.5% (11/315 errors out of the recorded prescriptions total) intervention ‐ The overall discrepancy rate was 0.68 per patient (SD 1.28); it was 1.49 (SD 1.66) in the pre‐phase and 0.15 (SD 0.46) in the post‐intervention phase (*p* < .001). ‐ Among the 84 unintentional medication discrepancies identified from the 489 medications surveyed, the most frequent medication error was ‘omission’ (56%). **Errors in new medication prescribing** ‐ 9.5%. **Reduction in patient harm**. − 14 (29%) of 49 patients (C) versus 5 (7%) of 74 (I) had at least 1 unintentional cause severe clinical deterioration discrepancy (*p* < .01) (Cohen's kappa, K = 0.447; *p* < .001). *From the medication reconciliation discrepancies combined in both arms combined*: 61% (51/84 error in total) caused severe discomfort. 18% (15/84 errors in total) caused moderate discomfort. 21% (18/84 errors in total) were unlikely to cause discomfort. *Errors as a percentage of the total charted prescriptions* ‐ 14.9% (73/489 errors out of the total recorded prescriptions) control. ‐ 2.2% (11/489 errors out of the total recorded prescriptions) intervention.
Pevnick J, et al. 2018^37^	Los Angeles, USA, ED, 01/07/2014 through 02/14/2014.	Three‐arm randomized controlled trial.	306 participants. Analysis completed on: 95 in the baseline control. 89 technician intervention. 94 pharmacist intervention.	Mondays through Thursdays from approximately 11 AM to 8 PM. Inclusion criteria were: ≥10 active chronic prescription medications in the electronic health record (EHR), history of acute myocardial infarction or congestive heart failure in the EHR problem list, admission from a skilled nursing facility (SNF), history of transplant, or active anticoagulant, insulin, or narrow therapeutic index medications.	Patients were excluded if they had previously been enrolled in the study, or if admitted to pediatric or trauma services or transplant services with pharmacists.	(C): physician. (I): pharmacist. (I):Pharmacist‐supervised pharmacy technician.	Medication discrepancies and severity.	**Medication errors during reconciliation** 69% (192/278 patients) experienced 1016 errors. Mean +/− SD Admission Medication History (AMH) errors per patient in the usual care, pharmacist and technician arms were 8.0 +/− 5.6, 1.4 +/− 1.9, and 1.5 +/− 2.1, respectively (*p* < .0001). **Reduction in patient harm.** *From the medication reconciliation discrepancies in the control and intervention arm separately*: *(all arms assessed together)* Pharmacist rater: 39% (399/1016 errors in total) caused significant harm. 60% (605/1016 errors in total) caused serious harm. 1% (12/1016 errors in total) caused life‐threatening. Physician rater: 62% (261/419 errors in total) caused significant harm. 37% (155/419 errors in total) caused serious harm. 1% (3/419 errors in total) caused life‐threatening harm. 69% (192/278 patients) experienced 1016 errors *Errors as a percentage of medication errors within each arm* ‐ 53.3% (760/1425 errors out of the recorded prescriptions total) control. ‐ 10% (133.5/1335 errors out of the recorded prescriptions total) technician intervention. ‐ 9.3% (131.6/1410 errors out of the recorded prescriptions total) pharmacist intervention. *Errors as a percentage of the total charted prescriptions* ‐ 18.2% (760/4170errors out of the total recorded prescriptions) control. ‐ 3.2% (133.5/4170 errors out of the total recorded prescriptions) technician intervention. ‐ 3.2% (131.6/4170errors out of the total recorded prescriptions) pharmacist intervention.
Sproul A, et al. 2018^33^	Canada, 2 site, ED, July, and August 2016 (2‐month period).	Prospective trial.	40 participants: Analysis completed on 39 participants (same group in the intervention and control arm).	Weekdays (Monday to Friday) within ED for whom nursing staff had completed a history.		(C): nurses. (I): trained pharmacy student.	Discrepancies and severity.	**Medication errors during reconciliation** 171 (C) errors versus 43 (I) errors, p = 0.006. Omissions 24 (C) versus 4 (I) p = 0.036. **‐ Errors in new medication prescribing** Incorrect medication 14 (C) versus 2 (I) p = 0.16. **Reduction in patient harm.** *From the medication reconciliation discrepancies in the control and intervention arm separately*: 56.1% (32/57 errors in total in the intervention group) and 59% (101/171 errors in total in the control group) unlikely to cause potential discomfort or clinical deterioration. 42.1% (24/57 errors in total in the intervention group) and 35.1% (60/171 errors in total in the control group) which could cause moderate discomfort or clinical deterioration. 1.8% (1/57 errors in total in the intervention group) and 5.8% (10/171 errors in total in the control group) potentially resulting in severe discomfort or clinical deterioration.
Arrison W, et al. 2020^34^	Georgia, USA, single site, ED, December 2018 through January 2019.	Single‐centre, retrospective, observational analysis.	204 patients: Control: 102. Intervention 102.	Presenting to a community hospital ED between 10:00 and 18:00.	Patients were excluded if all the following criteria were met unable to provide a medication history, have an unknown preferred pharmacy, and no other resources available to perform a medication history.	(C): nurse. (I): pharmacy technician.	Medication discrepancies and a number of high‐impact discrepancies.	**Medication errors during reconciliation** ‐ Medication history accuracy conducted by a pharmacy technician (94.1%) versus nurses (57.8%); *p* < .01. ‐ A total of seven discrepancies were found in the pharmacy technician group compared to 131 in the nursing group (*p* < .01). − 64% omissions. **Errors in new medication prescribing** − 45% 138 in total commission. **Reduction in patient harm.** *High impact discrepancies* 1 (I) versus 15 (C); (*p* < .01). *Errors as a percentage of medication errors within each arm* ‐ 17% (131/769 errors out of the recorded prescriptions total) control. ‐ 0.8% (7/903 errors out of the recorded prescriptions total) intervention. *Errors as a percentage of the total charted prescriptions* ‐ 7.8% (131/1672 errors out of the total recorded prescriptions) control. ‐ 0.4% (7/1672 errors out of the total recorded prescriptions) intervention
Do T, et al. 2021^35^	Cleveland, USA, single site community teaching hospital, ED, “pre‐medication history group” (patients admitted from January 1, 2017, through June 30, 2017) “post–medication history group” (those admitted from August 1, 2017, through February 28, 2018, excluding October).	Pre‐post(retrospective cohort study) study in an ED.	215 patients. Analysis completed on: 91 in the pre‐medication history group. 92 in the post‐medication history group.	Patients were included in the study if they were admitted through the main community teaching hospital ED and were taking 1 or more medications.	Patients were excluded if they were pregnant or less than 18 years of age. Patients were identified by using the ED electronic health record and the patient list maintained by the medication history program.	(C): usual MDT: nurses / physicians (I): pharmacy technicians	Medication accuracy and discrepancies.	**Medication errors during reconciliation** Accurate medication histories were obtained from 38% (C) and 70% (I) patients (*p* < .001), whereas accurate medication history was collected for 73% of medications used by patients in the (C) group and 93% in the (I) group (*p* < .001). 1773 medications reviewed. ‐ Within the 297 inaccurate medication histories identified, there were 345 errors: 268 (I) versus 77 (I).

Abbreviations: AMH, admission medication history; ADRs, adverse drug reactions; CI, confidence interval; C, control; EHR, electronic health record; ED, emergency department; I, intervention; OR, odds ratio; RR, relative risk; SNF, skilled nursing facility; SD, standard deviation.

### 3.1 Risk of bias assessment

**FIGURE 2:**
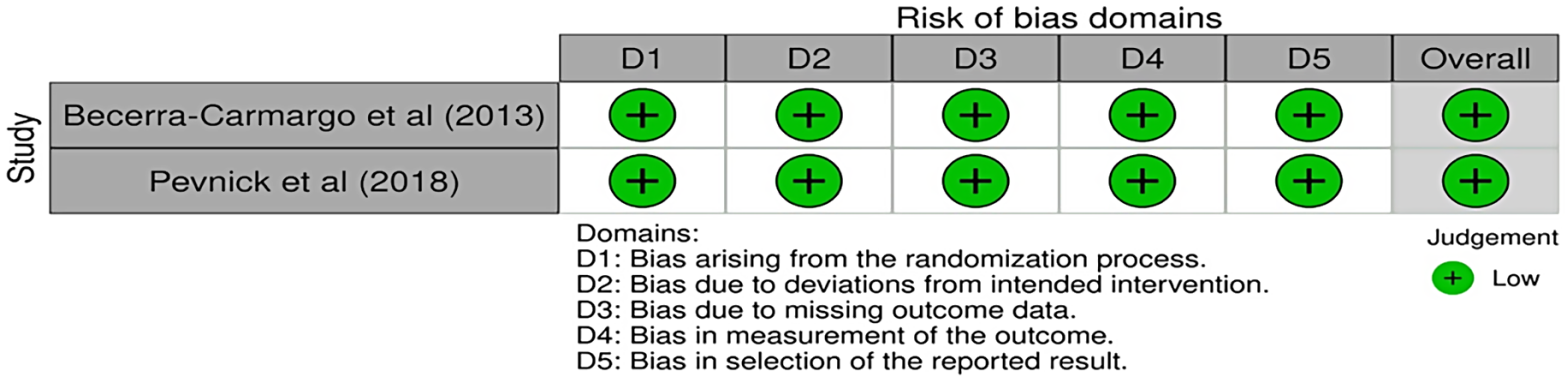
Cochrane Risk of bias tool (ROB2® Cochrane, UK) applied to the randomized controlled trials. The two studies included were randomized controlled trials.

**FIGURE 3:**
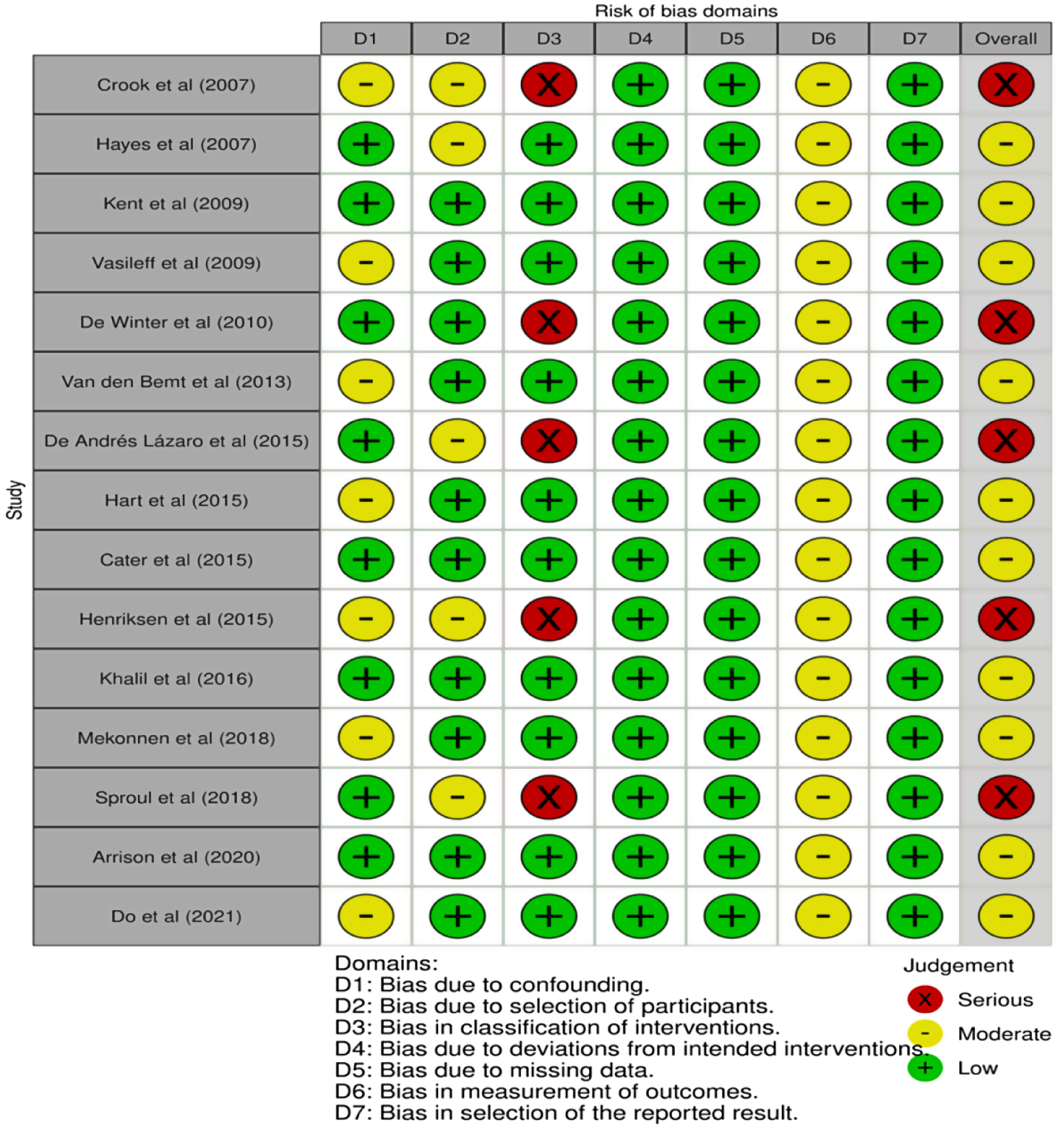
Risk Of Bias In Non‐randomized Studies of Interventions (ROBIN‐I Cochrane, UK) tool applied to the non‐randomized controlled trials. The fifteen studies included were randomized controlled trials.

Two RCTs^36^, ^37^ both had a low risk of bias across five domains; see Figure 2. For the 15 NRCTs,^21^, ^22^, ^23^, ^24^, ^25^, ^26^, ^27^, ^28^, ^29^, ^30^, ^31^, ^32^, ^33^, ^34^, ^35^ seven of the studies^21^, ^24^, ^26^, ^29^, ^30^, ^32^, ^35^ may have been affected by confounding variables. One of which was patient recruitment into the studies, as this was not reflective of all service users admitted into the hospital setting as some only included elderly patients or those who were prescribed two or more medications.^21^, ^24^, ^26^, ^29^, ^30^, ^32^, ^35^ Selection bias could have moderately affected five of the studies^21^, ^22^, ^28^, ^30^, ^33^ owing to changes occurring with the inclusion and exclusion criteria once the studies had commenced. A moderate level of outcome bias was noted for all 15 NRCTs owing to unclear and limited blinding of the participants. Outcome assessors may also have caused bias by knowing which arm a participant had been assigned. Five of the NRCTs^21^, ^25^, ^28^, ^30^, ^33^ demonstrated a high risk of bias since assignment to the intervention arm was not blinded. See Figures 2 and 3 for a pictorial assessment of bias across the studies.

### 3.2 Systematic review outcomes

#### 3.2.1 Medication errors during reconciliation including omissions and commissions

**FIGURE 4:**
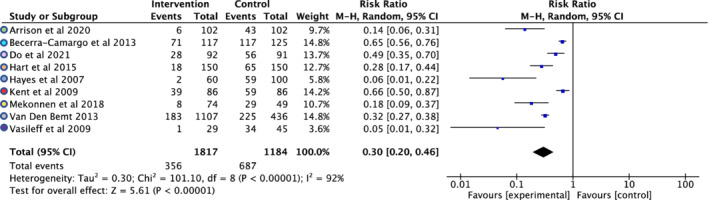
Forest plot for nine studies^22^, ^23^, ^24^, ^26^, ^29^, ^32^, ^34^, ^35^, ^36^ measuring medication errors. Medication errors for both arms were inclusive of any transcription errors compared to medication taken prior to admission to the acute or emergency medicine department.

**FIGURE 5:**
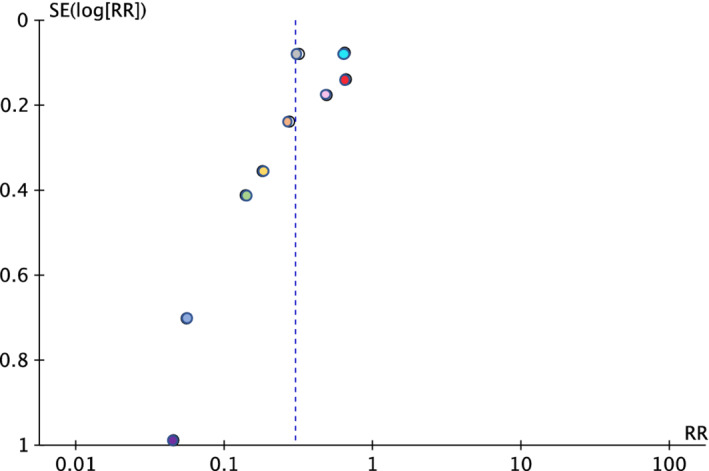
Funnel plot of the effects of a pharmacy intervention versus the control arm for providing a medication history on admission to an acute or emergency medicine department, for nine studies.^22^, ^23^, ^24^, ^26^, ^29^, ^32^, ^34^, ^35^, ^36^ Each dot represents one study. A positive outcome would be the equal distribution of studies, with those which were larger (with more power) being at the top. The x‐axis shows the 95% confidence interval (CI) of the risk ratio. For this review, this is the risk of a medication error happening in the pharmacy intervention group compared to the control group. The *y* axis shows the standard error of the effect measure.

Medication errors on admission were measured at the point of reconciliation after the initial history was recorded. These included results that would allow the risk reduction to be calculated for nine of the 17 studies including one out of two RCTs and eight out of 15 NRCTs. A medication error was defined as any unintentional changes in prescriptions (from primary care into secondary care) on a patient's physical or electronic medication chart on admission and was inclusive of commissions (unintentional new medications) or omissions (unintentionally missed medications). Figure 4 shows the forest plot and Figure 5 shows the funnel plot for this outcome across included studies with the calculated risk ratio. Whilst the Chi^2^ test showed statistical significance, the data were highly heterogeneous. The *I* ^2^ value was reported at 92% and showed considerable variation across studies, owing to study heterogeneity. The funnel plot indicates there may have been publication bias.

#### 3.2.2 Inappropriate dose adjustments for patient characteristics on admission

Dose adjustments were not reported as an investigated outcome in both the control and intervention arms within any of the studies.

#### 3.2.3 Reduction in patient harm

Reduction in patient harm was extracted as a primary co‐outcome from the studies using pre‐defined criteria from each study. The outcome was only reported in seven of the 17 included studies (Table 3) (both RCTs and five out of 15 NRCTs). The reported outcomes for the severity of the transcription errors were reported on a range of scales that differed between the studies. For convenience, the potential for harm could be summarized into three categories, to enable comparisons between the studies as severe discomfort, moderate discomfort, or unlikely to cause any discomfort.

**TABLE 3:** Seven studies showing the severity of prescription errors and the severity scale used

Studies	Criteria definitions	Who determined potential harm	Patient numbers	Overall medicine errors (numbers and percentage)	Number of reductions in potential patient harm events
Vasileff HM, et al. 2009^24^	Local pre‐defined criteria 0 = Nil impact (No impact on health outcomes, short or long term). 1 = Minor impact (Minor impact on health outcomes). 2 = Significant impact (Potentially significant impact on health outcomes). 3 = Very significant impact (Without intervention, potentially severe impact on health outcomes). 4 = Life‐saving impact (Potentially life‐saving impact on health outcomes).	The clinical significance was assessed by a multidisciplinary panel, comprising three hospital pharmacists, three doctors, one academic pharmacist, and the pharmacy researcher. Who independently assigned a level of clinical significance to each intervention according to predefined criteria. The mean level of clinical significance was calculated and rounded to the nearest clinical significance category. The degree of inter‐rater reliability was assessed by calculating the gamma statistic for each possible pairing of raters.	74 participants: 45 in the control. 29 in the intervention.	‐ Patient with 1 or more error: 76% (C) versus 3.3% (I) unintentional discrepancies. *Author contacted for further information, but no response received*.	*From the medication reconciliation discrepancies in the control arm*: 6% (7/111 errors in total) very significant impact. 52% (58/111 errors in total) significant impact. 40% (44/111 errors in total) minor impact. 2% (2/111 errors in total) no impact.
Becerra‐Camargo J, et al. 2013^36^	Adapted from Cornish et al, 2005^39^ Class 1: discrepancies unlikely to cause potential discomfort or clinical deterioration. Class 2: discrepancies that could cause moderate discomfort or clinical deterioration. Class 3: discrepancies potentially resulting in severe discomfort or clinical deterioration.	The clinical severity of medication discrepancies was independently assessed by two clinical pharmacists blinded to the patient data collection forms; they classified each type of medication discrepancy according to its potential to cause harm. Disagreements were resolved by discussion and consensus was reached for all discrepancies.	270 participants. Analysis completed on: Control 125. Intervention 117.	*Author contacted for further information, but no response received*. **Errors as a percentage of the total charted prescriptions:** ‐ 45.2% (528/1169 errors out of the total recorded prescriptions) control ‐ 24.2% (283/1169 errors out of the total recorded prescriptions) intervention	*From the medication reconciliation discrepancies combined in both arms combined*: 33.4% (271/811 errors in total) discrepancies unlikely to cause potential discomfort or clinical deterioration. 42.7% (346/811 errors in total) discrepancies that could cause moderate discomfort or clinical deterioration. 23.9% (194/811 errors in total) discrepancies potentially resulting in severe discomfort or clinical deterioration.
Cater SW, et al. 2015^27^	Adapted from NCC MERP, 2001^38^ 0 = Insignificant error. 1 = Minimal error. 2 = Major error.	One study author classified all medication discrepancies by type and performed initial error ratings. A second author and dual board‐certified Internal Medicine an Emergency Physician performed a blinded, independent review of error ratings for a validation set of 25 cases. Inter‐rater reliability resulted in a high Cohen's value.	188 participants: Control 75. Intervention 113.	**Errors as a percentage of medication errors within each arm:** ‐ Data not available for the number of prescriptions within each arm. **Errors as a percentage of the total charted prescriptions:** ‐ 44.9% (661/1473 errors out of the total recorded prescriptions) control ‐ 61.4% (905/1473 errors out of the total recorded prescriptions) intervention.	*From the medication reconciliation discrepancies in the control and intervention arm separately*: 35.7% (126/353 errors in total in the intervention group) and 34.7% (79/228 errors in total in the control group) insignificant errors. 34.0% (120/353 errors in total in the intervention group) and 38.6% (88/228 errors in total in the control group) minimal errors. 30.3% (107/353 errors in total in the intervention group) and 26.8% (61/228 errors in total in the control group) insignificant errors.
Khalil V, et al. 2016^31^	Adapted from The Society of Hospital Pharmacists of Australia, 2013^41^ ‐ Extreme and high ‐ Moderate ‐ Low.	The severity of all errors was then rated by a blinded consultant physician and an independent senior pharmacist according to the standardized matrix and recorded for analysis.	110 participants Control 54 Intervention 56	**Errors as a percentage of medication errors within each arm:** ‐ 43% (238/554 errors out of the recorded prescriptions total) control. ‐ 4.9% (29/595 errors out of the recorded prescriptions total) intervention. **Errors as a percentage of the total charted prescriptions:** ‐ 20.7% (238/1149 errors out of the total recorded prescriptions) control. ‐ 2.5% (29/1149 errors out of the total recorded prescriptions) intervention.	*From the medication reconciliation discrepancies in the control and intervention arm separately*: 0% (0/595 errors in total in the intervention group) and 17.5% (97/554 errors in total in the control group) extreme and high harm. 1% (6/595 errors in total in the intervention group) and 11.6% (64/554 errors in total in the control group) moderate harm. 3.9% (23/595 errors in total in the intervention group) and 13.9% (77/554 errors in total in the control group) low harm.
Mekonnen AB, et al. 2018^32^	Adapted from Cornish et al, 2005^39^ Class 1: discrepancies unlikely to cause potential discomfort or clinical deterioration. Class 2: discrepancies that could cause moderate discomfort or clinical deterioration. Class 3: discrepancies potentially resulting in severe discomfort or clinical deterioration.	The potential clinical severity of medication discrepancies was judged by a consensus between a clinical pharmacist/physician. There was a moderate level of agreement (Cohen's kappa) among evaluators in judging the potential clinical impact of medication discrepancies.	123 patients: Control: 49. Intervention 74.	**Errors as a percentage of medication errors within each arm:** ‐ 42% (73/174 errors out of the recorded prescriptions total) control. ‐ 3.5% (11/315 errors out of the recorded prescriptions total) intervention. **Errors as a percentage of the total charted prescriptions:** ‐ 14.9% (73/489 errors out of the total recorded prescriptions) control. ‐ 2.2% (11/489 errors out of the total recorded prescriptions) intervention.	*From the medication reconciliation discrepancies combined in both arms combined*: 61% (51/84 error in total) caused severe discomfort. 18% (15/84 errors in total) caused moderate discomfort. 21% (18/84 errors in total) were unlikely to cause discomfort.
Pevnick J, et al. 2018^37^	Adapted from Bates et al, 1995^42^ ‐ Significant. ‐ Serious. ‐ Life‐threatening.	Expert pharmacists assigned the classifications, a second pharmacist reviewed these classifications. A physician adjudicated disagreements. The study arms were not masked.	306 participants. Analysis completed on: 95 in the baseline control. 89 technician intervention. 94 pharmacist intervention.	69% (192/278 patients) experienced 1016 errors: **Errors as a percentage of medication errors within each arm:** ‐ 53.3% (760/1425 errors out of the recorded prescriptions total) control. ‐ 10% (133.5/1335 errors out of the recorded prescriptions total) technician intervention. ‐ 9.3% (131.6/1410 errors out of the recorded prescriptions total) pharmacist intervention. **Errors as a percentage of the total charted prescriptions:** ‐ 18.2% (760/4170errors out of the total recorded prescriptions) control. ‐ 3.2% (133.5/4170 errors out of the total recorded prescriptions) technician intervention. ‐ 3.2% (131.6/4170errors out of the total recorded prescriptions) pharmacist intervention.	*From the medication reconciliation discrepancies in the control and intervention arms separately (all arms assessed together)*: Pharmacist rater: 39% (399/1016 errors in total) caused significant harm. 60% (605/1016 errors in total) caused serious harm. 1% (12/1016 errors in total) caused life‐threatening. Physician rater: 62% (261/419 errors in total) caused significant harm. 37% (155/419 errors in total) caused serious harm. 1% (3/419 errors in total) caused life threatening harm.
Sproul A, et al. 2018^33^	Adapted from Cornish et al, 2005^39^ Class 1: discrepancies unlikely to cause potential discomfort or clinical deterioration. Class 2: discrepancies that could cause moderate discomfort or clinical deterioration. Class 3: discrepancies potentially resulting in severe discomfort or clinical deterioration	A panel of practitioners (one pharmacist, one physician, and one nurse) who were not involved in obtaining or comparing the medication histories independently determined the potential severity of each error.	40 participants: Analysis completed on 39 participants (same group in the intervention and control arm).	171 (C) errors versus 43 (I) errors, *p* = .006. *Author contacted for further information, but no response received*.	*From the medication reconciliation discrepancies in the control and intervention arm separately*: 56.1% (32/57 errors in total in the intervention group) and 59% (101/171 errors in total in the control group) unlikely to cause potential discomfort or clinical deterioration. 42.1% (24/57 errors in total in the intervention group) and 35.1% (60/171 errors in total in the control group) that could cause moderate discomfort or clinical deterioration. 1.8% (1/57 errors in total in the intervention group) and 5.8% (10/171 errors in total in the control group) potentially resulting in severe discomfort or clinical deterioration.

*Note*: The severity scales used were defined within the table.

There was considerable heterogeneity in how the severity of the medication errors was rated. Within seven studies, one used a local defined scale.^24^ One of the study scales^27^ appeared to use a scale validated by NCC MERP, 2001,^38^ whilst the others differed using either their own or other published scales based on the potential for harm summarized into three categories of severe discomfort, moderate discomfort or unlikely to cause any discomfort. Three studies used the severity scales from Cornish et al, 2005,^32^, ^33^, ^36^ two studies used two different national severity scales^24^, ^34^ and one study used a scale by Bates, et al, 1995.^37^ The scale from Bates et al, 1995 was used by Pevnick J, et al. 2018^37^ and included an additional level noted as life‐threatening errors. The studies that used the severity scale by Cornish et al, 2005^39^ could not be compared, as in some studies the figures reported a combined value for both the control and intervention arms, preventing a risk reduction analysis.

Only two studies had an independent group of staff assessing the severity of the errors.^24^, ^33^ Three studies^27^, ^31^, ^36^ blinded the assessors when grouping the severity of the interventions, but it is unclear if the groups of assessors were authors in the studies. Cater SW, et al. 2013^27^ assessed the severity with a second check by a blinded physician but this was only for a sub‐sample of 25 cases. Mekonnen AB, et al (2018) and Pevnick et al (2018) included the use of staff that was not blinded during the severity rating and who may have been a part of the studies.^32^, ^37^

#### 3.2.4 Speed of processing patients through acute and emergency services

One out of 17 included studies reported the time taken to complete the medication history within both arms (1 NRCT)^30^ and owing to the limited number of studies providing this outcome, it was not possible to calculate hazard ratios. The single study assessing this outcome described it taking an average of 44 min (range 15–150 min) for the pharmacy intervention arm to complete the medication history compared to the 9.6 min (range 6–13 min) in the control arm.

#### 3.2.5 The effect of Pharmacy staff experience on the pharmacy service provided

None of the studies reported the seniority of pharmacy professionals.

#### 3.2.6 The effect of Multimorbidity and polypharmacy on patients' risk of experiencing medication errors

None of the studies reported the impact of multi‐morbidity or polypharmacy.

## 4 DISCUSSION

To the authors' knowledge, this is the first systematic review assessing the impact of pharmacy services across both acute and emergency medicine settings. The review examined all studies that included the accuracy of medication histories and reconciliations on admission to the hospital comparing a control (non‐pharmacy arm) with an intervention (pharmacy arm). The review aimed to provide an evidence base for service developments within Acute Medicine or Emergency departments (ED).

The meta‐analysis indicated that the implementation of a pharmacy service within acute medical or emergency departments resulted in a significant reduction of medication errors by approximately 70%. This is consistent with other published data where the presence of pharmacy services resulted in fewer prescribing errors.^10^, ^11^, ^13^ The present results are also concordant with a NICE commissioned systematic review (2007)^40^ which concluded that an investment of £2000 in pharmacist‐led medication reconciliation in hospitals saved £3000 per 1000 medications reviewed.

All nine of the studies included in the statistical analysis within the present review demonstrated a significant reduction in medication errors, which was one of the primary outcomes of the systematic review. The reasons for this were not explored but medication histories and reconciliations may have been more accurate as they reflected the processes and experience of the pharmacy teams who would normally conduct this as a key aspect of their day‐to‐day role.^21^ Whilst all studies reported similar findings, only one of the nine reported studies was a RCT. There was a high level of heterogeneity associated with the NRCTs owing to; differences in the inclusion criteria; variability in staff members completing the medication history process in each arm; differences in healthcare centers between different countries; the resources available in the units to support this task^36^; the number of sites investigated during the individual studies and the criteria used to define a discrepancy.^24^ These factors and the lack of blinding to the intervention or the assessment of medication errors prevent these studies from providing a definitive answer to the clinical efficacy of the intervention. It is also unclear how representative the recruited population might be of patients attending hospitals acutely, especially in terms of multi‐morbidity and polypharmacy. Whilst medication dose adjustments were reported in one study^21^ it was unclear whether these adjustments were to be due to patient factors such as the patient's age, weight, and organ function on admission.

Seven studies assessed the potential harm that may have arisen from medication errors. There were limitations associated with the severity of harm being assessed, as some were not assessed by an independent team, thus increasing the risk of bias within the studies.^27^, ^31^, ^32^, ^36^, ^37^ It is unclear whether the severity of the medicine error may have been graded differently on admission, compared to the actual harm the error might have caused if left uncorrected. For example, the omission of insulin may have severe consequences that would only become apparent after a few missed doses, in comparison to noting the discrepancy initially on presentation.^24^ The included studies indicated that some errors, if not corrected on admission, may have persisted throughout the admission, leading to an error on return into primary care. For example, Vasileff, et al., 2009 noted that 13% of discrepancy errors had not been resolved at the point of patients' discharge back into primary care.^24^ If not corrected these could result in errors compounding back through to the primary care services.^21^, ^27^, ^28^, ^32^

Secondary review outcomes aimed to examine the speed of clerking patients, however, this information was not collected or reported in many of the included studies. Whilst the time taken to complete a medication history was averaged in some of the studies, it is unclear if the same process or number of resources were used across these studies. As a result, it was difficult to compare the average time taken to complete medication histories for each control and intervention group. The seniority of the staff (in terms of years qualified and courses completed) was not compared as this was not reported in any of the included studies. A systematic review and meta‐analysis conducted by Choi and Kim 2019 reported no difference in the performance between technicians or pharmacists completing medication reconciliations within ED for five of the studies they reviewed.^10^ However, the subgroup analysis within their study did not comment on whether the complexity of cases had been matched across groups.

No studies commented on the impact of multimorbidity or polypharmacy, which would have been helpful to understand if future pharmacy services should be targeted at more complex medicine regimes, given our aging population.

### 4.1 Strengths and weaknesses

The strengths of this review included the similarity of the results of the studies. Each described the benefit of a pharmacy team reducing medication errors within an emergency department and during transitions of care.^10^, ^11^, ^13^ This was the largest review examining the existing published data around pharmacy services for patients admitted to either the acute or emergency departments, performed using standard methodologies with a protocol completed and published prior to review commencement.

The limitations of this review were that study heterogeneity and differences in the metrics that were reported resulted in a narrative review for many studies, with a statistical analysis only possible in a subgroup rather than a quantitative meta‐analysis. Some of the smaller studies included within this review may have bias associated with their results, as effect size can be overestimated in studies that include smaller populations. Studies may have been affected by confounding variables for example the recruitment of patients was mainly during the day and on weekdays rather than on a weekend or at night. For some studies, this had been noted in the inclusion criteria^22^, ^23^, ^24^, ^25^, ^28^, ^29^, ^31^, ^32^, ^33^, ^34^, ^37^ but there was no comparison or reference to the recruitment of participants out‐of‐hours. The statistical subgroup analysis of the studies was performed on less than 50% of the articles consisting of 3001 patients.

A subgroup analysis was planned to explore if there are certain categories of medications for which errors are experienced more commonly or where medications are missed potentially owing to their perceived importance by patients and staff members. For example, a review of whether topical preparations or medications are used when required as opposed to regularly, is less likely to be noted within a medication history.^21^, ^24^, ^25^ This could not be assessed based on the reported outcomes.

One of the main limitations of this review is that all studies included were set outside of the UK, either within Europe or internationally. As a result, the generalisability of these results to the UK NHS may be limited, owing to the data being reported from places that may have private healthcare systems. Further studies are needed that assess UK health settings prior to service modification.

The studies presented within this systematic review indicate that medication reconciliation services provided by pharmacy staff decrease the number of medication discrepancies. However, there are several uncertainties that remain which would benefit from further research. The full benefit of a pharmacy service out of hours is unclear. It is also unclear whether benefits would be greater if targeted, for example, to those with complex care needs or polypharmacy. As highlighted in a recent report by Ridge., 2021^4^ the pharmacy workforce might be best placed to support medication optimization and deprescribing within hospitals. It would be beneficial to examine services that move from following up errors retrospectively, to proactively, preventing these within both the emergency and acute departments.^22^

## 5 CONCLUSION

The studies included within this systematic review consistently indicate that pharmacy services based within the acute and emergency medicine departments in hospitals are associated with fewer medication errors. However, these results should be interpreted with caution as studies were affected by bias, heterogenous in design, and often included unblinded assessments of efficacy. All studies were set outside of the UK and differences in healthcare models might impact the results, meaning that studies might need to be replicated in specific UK settings to determine whether the model of care should be adjusted. Further studies are needed to understand the health and economic impact of deploying a pharmacy service in acute medical settings. However, to date, the evidence indicates that pharmacy services providing medicine reconciliation at the point of admission to the hospital reduce medicines errors and that these services should be assessed in more detail.

Conventionally, pharmacists have not been deployed within emergency departments, but this is changing. Patients are spending considerable time within ED, and it is recognized that patients using ED are increasingly complex with significant polypharmacy. There are known patient harm from missing regular medications, as described within this systematic review. Currently, there is variation in the type of pharmacy services that exist nationally within Emergency and Acute Medicine departments, and often limited funding is available for these services. Future research should assess best practices and service models which improve flow and increase patient safety, including pharmacy team reviews earlier in the admission, focusing services on more complex patients, and out‐of‐hours work.

## FUNDING INFORMATION

EP received funding from the National Institute of Health Research (NIHR) Clinical Research Scholar (CRS) Award Programme 2020 to conduct the attached systematic review.

## CONFLICT OF INTEREST

ES reports grants from HDR‐UK, during the conduct of the study; grants from Medical Research Council, grants from NIHR, grants from Wellcome Trust, grants from British Lung Foundation, and grants from Alpha 1 Foundation, outside the submitted work.

## ETHICS APPROVAL STATEMENT

As a systematic review of published literature, no specific ethical approvals were needed.

## Supporting information


Appendix S1

## Data Availability

The data that support the findings of this study are available on request from the corresponding author.
