# Supplementary material for: What is the evidence that a pharmacy team working in an acute or emergency medicine department improves outcomes for patients: A systematic review
Source: Pharmacol Res Perspect. 2022 Sep 14;10(5):e01007. doi: 10.1002/prp2.1007 (PMC9471999; doi:10.1002/prp2.1007)
Supplement: Supplementary file 1 — Appendix S1 [file PRP2-10-e01007-s001.docx]

Supplement

Databases included the Cochrane Database of Systematic Reviews (CDSR) (1995 to 08/11/20220), MEDLINE (Ovid) (1946 to 14/11/2020), Embase (Ovid) (1974 to 14/11/2020), CINAHL Plus (EBSCO) (1937 to 08/11/2020), MEDLINE in Process (Ovid) (1946 to 15/11/2020), Cochrane Central Register of Controlled Trials (CENTRAL) (1996 to 29/11/2020), PsycInfo (Ovid) (1806 to 14/11/2020), Healthcare Management Information Consortium (HMIC) database (1979 to 15/11/2020), Web of Science (1900 to 29/11/2020). One trial register ClinicalTrials.gov (15/11/2020) was searched. The International Clinical Trials Registry Platform (ICTRP) ([www.who.int/ictrp/en/](http://www.who.int/ictrp/en/)) was searched on 12/01/2022 as initially the website could not be searched due to the Covid-19 pandemic generating high traffic through the website.

Table of Contents

[Medline – via HDAS NICE (OVID) 14/11/2020 5](#_Toc95992624)

[Embase – via HDAS NICE 14/11/2020 6](#_Toc95992625)

[PsycInfo– via HDAS NICE 14/11/2020 7](#_Toc95992626)

[Medline in process -via UoB (OVID): 15/11/2020 8](#_Toc95992627)

[CINAHL plus – via UoB (EBSCO) 08/11/2020 9](#_Toc95992628)

[Healthcare Management of Information Consortium (HMIC) – via UoB 15/11/2020 13](#_Toc95992629)

[Clinical Trails.Gov 15/11/2020 14](#_Toc95992630)

[International Clinical Trials Registry Platform (ICTRP) 12/01/2022 15](#_Toc95992631)

[CENTRAL and CDSR using Medline search (via Cochrane) 29/11/2020 16](#_Toc95992632)

[Web of Science 29/11/2020 forward citation completed 25/05/2021 17](#_Toc95992633)

# Medline – via HDAS NICE (OVID) 14/11/2020

1. Medline exp "MEDICATION RECONCILIATION"/ (1,156)
2. Medline (''medication reconciliation'').ti,ab (1,589)
3. Medline (''medical reconciliation'').ti,ab (789)
4. Medline (''drug history'').ti,ab (35,168)
5. Medline (''medication history'').ti,ab (16,344)
6. Medline (1 OR 2 OR 3 OR 4 OR 5) (50,556)
7. Medline (''pharmac*).ti,ab (127,527)
8. Medline exp "PHARMACY SERVICE, HOSPITAL"/ (11,707)
9. Medline exp PHARMACISTS/ (17,036)
10. Medline exp "PHARMACY TECHNICIANS"/ (747)
11. Medline (''pre-registration pharmacist*'').ti,ab (37)
12. Medline (''pharmacy intervention'').ti,ab (3,072)
13. Medline (7 OR 8 OR 9 OR 10 OR 11 OR 12) (134,096)
14. Medline exp "EMERGENCY SERVICE, HOSPITAL"/ (80,083)
15. Medline exp "HOSPITAL DEPARTMENTS"/ (186,482)
16. Medline exp "EMERGENCY MEDICINE"/ (13,907)
17. Medline exp "EMERGENCY RESPONDERS"/ (12,675)
18. Medline (''emergency department*'').ti,ab (102,804)
19. Medline (''emergency medicine*'').ti,ab (20,648)
20. Medline (''accident AND emergency'').ti,ab (8,233)
21. Medline (''emergency care'').ti,ab (76,936)
22. Medline (''acute medicine unit'').ti,ab (1,138)
23. Medline (''acute medicine care'').ti,ab (5,451)
24. Medline (''acute medical unit*'' OR AMU).ti,ab (13,014)
25. Medline (''acute admissions unit*'' OR AMU).ti,ab (4,336)
26. Medline (''medical assessment unit*'' OR AMU).ti,ab (9,031)
27. Medline (''acute assessment unit*'').ti,ab (5,658)
28. Medline (''acute medical ward*'').ti,ab (2,645)
29. Medline (''acute planning unit*'').ti,ab (856)
30. Medline (''combined admissions unit*'').ti,ab (364)
31. Medline (''combined assessment unit*'').ti,ab (1,980)
32. Medline (''medical admissions unit*'').ti,ab (3,681)
33. Medline (''rapid assessment medical unit*'').ti,ab (269)
34. Medline (14 OR 15 OR 16 OR 17 OR 18 OR 19 OR 20 OR 21 OR 22 OR 23 OR 24 OR 25 OR 26 OR 27 OR 28 OR 29 OR 30 OR 31 OR 32 OR 33) (338,070)
35. Medline (6 AND 13 AND 34) (641)

# Embase – via HDAS NICE 14/11/2020

1. EMBASE exp "MEDICATION THERAPY MANAGEMENT"/ (11,637)
2. EMBASE (''medication reconciliation'').ti,ab (3,250)
3. EMBASE (''medical reconciliation'').ti,ab (42)
4. EMBASE (''drug history'').ti,ab (1,959)
5. EMBASE (''medication history'').ti,ab (3,101)
6. EMBASE (36 OR 37 OR 38 OR 39 OR 40) (16,826)
7. EMBASE (''pharmac*).ti,ab (1,134,198)
8. EMBASE exp "HOSPITAL PHARMACY"/ (14,106)
9. EMBASE exp PHARMACIST/ (80,362)
10. EMBASE exp "PHARMACY TECHNICIAN"/ (2,156)
11. EMBASE (''pre-registration pharmacist*'').ti,ab (74)
12. EMBASE (''pharmacy intervention'').ti,ab (278)
13. EMBASE exp "CLINICAL PHARMACY"/ (11,082)
14. EMBASE (42 OR 43 OR 44 OR 45 OR 46 OR 47 OR 48) (1,161,693)
15. EMBASE exp "HOSPITAL EMERGENCY SERVICE"/ (5,506)
16. EMBASE exp "EMERGENCY WARD"/ (152,942)
17. EMBASE exp "EMERGENCY MEDICINE"/ (42,386)
18. EMBASE (''emergency department*'').ti,ab (146,891)
19. EMBASE (''emergency medicine*'').ti,ab (23,626)
20. EMBASE (''accident AND emergency'').ti,ab (11,368)
21. EMBASE exp "EMERGENCY CARE"/ (47,783)
22. EMBASE (''emergency care'').ti,ab (11,913)
23. EMBASE (''acute medicine unit'').ti,ab (34)
24. EMBASE (''acute medicine care'').ti,ab (3)
25. EMBASE (''acute medical unit*'' OR AMU).ti,ab (1,787)
26. EMBASE (''acute admissions unit*'' OR AMU).ti,ab (1,418)
27. EMBASE (''medical assessment unit*'' OR AMU).ti,ab (1,598)
28. EMBASE (''acute assessment unit*'').ti,ab (65)
29. EMBASE (''acute medical ward*'').ti,ab (338)
30. EMBASE (''combined assessment unit*'').ti,ab (2)
31. EMBASE (''medical admissions unit*'').ti,ab (182)
32. EMBASE (''rapid assessment medical unit*'').ti,ab (4)
33. EMBASE (50 OR 51 OR 52 OR 53 OR 54 OR 55 OR 56 OR 57 OR 58 OR 59 OR 60 OR 61 OR 62 OR 63 OR 64 OR 65 OR 66 OR 67) (284,035)
34. EMBASE (41 AND 49 AND 68) (726)

# PsycInfo– via HDAS NICE 14/11/2020

1. PsycINFO (''medication reconciliation'').ti,ab (153)
2. PsycINFO (''medical reconciliation'').ti,ab (125)
3. PsycINFO (''drug history'').ti,ab (10,916)
4. PsycINFO (''medication history'').ti,ab (4,874)
5. PsycINFO (70 OR 71 OR 72 OR 73) (15,120)
6. PsycINFO (''pharmac*).ti,ab (13,361)
7. PsycINFO exp PHARMACISTS/ (1,501)
8. PsycINFO (''pre-registration pharmacist*'').ti,ab (9)
9. PsycINFO (''pharmacy intervention'').ti,ab (424)
10. PsycINFO (''pharmacy technician*'').ti,ab (62)
11. PsycINFO (75 OR 76 OR 77 OR 78 OR 79) (13,392)
12. PsycINFO exp "EMERGENCY MEDICINE"/ (332)
13. PsycINFO (''emergency department*'').ti,ab (9,637)
14. PsycINFO (''emergency medicine*'').ti,ab (1,309)
15. PsycINFO (''accident AND emergency'').ti,ab (794)
16. PsycINFO (''emergency care'').ti,ab (9,286)
17. PsycINFO (''acute medicine unit'').ti,ab (91)
18. PsycINFO (''acute medicine care'').ti,ab (626)
19. PsycINFO (''acute medical unit*'' OR AMU).ti,ab (1,285)
20. PsycINFO (''acute admissions unit*'' OR AMU).ti,ab (373)
21. PsycINFO (''medical assessment unit*'' OR AMU).ti,ab (1,648)
22. PsycINFO (''acute assessment unit*'').ti,ab (787)
23. PsycINFO (''acute medical ward*'').ti,ab (464)
24. PsycINFO (''acute planning unit*'').ti,ab (186)
25. PsycINFO (''combined admissions unit*'').ti,ab (43)
26. PsycINFO (''combined assessment unit*'').ti,ab (350)
27. PsycINFO (''medical admissions unit*'').ti,ab (391)
28. PsycINFO (''rapid assessment medical unit*'').ti,ab (22)
29. PsycINFO (81 OR 82 OR 83 OR 84 OR 85 OR 86 OR 87 OR 88 OR 89 OR 90 OR 91 OR 92 OR 93 OR 94 OR 95 OR 96 OR 97) (19,939)
30. PsycINFO (74 AND 80 AND 98) (17)

# Medline in process -via UoB (OVID): 15/11/2020

1. exp Medication Reconciliation/ 1174
2. ''medication reconciliation''.ti,ab. 1352
3. ''medical reconciliation''.ti,ab. 13
4. ''drug history''.ti,ab. 1031
5. ''medication history''.ti,ab. 1410
6. 1 or 2 or 3 or 4 or 5 4149
7. ''pharmac*.ti,ab. 744726
8. exp Pharmacy Service, Hospital/ 11718
9. exp Pharmacists/ 17103
10. exp Pharmacy Technicians/ 749
11. ''pre-registration pharmacist*''.ti,ab. 16
12. ''pharmacy intervention''.ti,ab. 125
13. 7 or 8 or 9 or 10 or 11 or 12 750578
14. exp Emergency Service, Hospital/ 80423
15. exp Hospital Departments/ 185856
16. exp Emergency Medicine/ 13965
17. exp Emergency Responders/ 12702
18. ''emergency department*''.ti,ab. 91772
19. ''emergency medicine*''.ti,ab. 14135
20. (''accident and emergency'').ti,ab. 8257
21. ''emergency care''.ti,ab. 8818
22. ''acute medicine unit''.ti,ab. 17
23. ''acute medicine care''.ti,ab. 2
24. (''acute medical unit*'' or AMU).ti,ab. 1300
25. (''acute admissions unit*'' or AMU).ti,ab. 1069
26. (''medical assessment unit*'' or AMU).ti,ab. 1143
27. ''acute assessment unit*''.ti,ab. 29
28. ''acute medical ward*''.ti,ab. 217
29. ''acute planning unit*''.ti,ab. 0
30. ''combined admissions unit*''.ti,ab. 0
31. ''combined assessment unit*''.ti,ab. 3
32. ''medical admissions unit*''.ti,ab. 80
33. ''rapid assessment medical unit*''.ti,ab. 2
34. 14 or 15 or 16 or 17 or 18 or 19 or 20 or 21 or 22 or 23 or 24 or 25 or 26 or 27 or 28 or 29 or 30 or 31 or 32 or 33 272107
35. 6 and 13 and 34 486

# CINAHL plus – via UoB (EBSCO) 08/11/2020

S39 S6 AND S13 AND S38 Expanders - Apply equivalent subjects

Search modes - Boolean/Phrase Interface - EBSCOhost Research Databases

Search Screen - Advanced Search

Database - CINAHL Plus 162 Edit S39

S38 S14 OR S15 OR S16 OR S17 OR S18 OR S19 OR S20 OR S21 OR S22 OR S23 OR S24 OR S25 OR S26 OR S27 OR S28 OR S29 OR S30 OR S31 OR S32 OR S33 OR S34 OR S35 OR S36 OR S37 Expanders - Apply equivalent subjects

Search modes - Boolean/Phrase Interface - EBSCOhost Research Databases

Search Screen - Advanced Search

Database - CINAHL Plus 121,264 Edit S38

S37 "rapid assessment medical unit*" Expanders - Apply equivalent subjects

Search modes - Boolean/Phrase Interface - EBSCOhost Research Databases

Search Screen - Advanced Search

Database - CINAHL Plus 4 Edit S37

S36 "medical admissions unit*" Expanders - Apply equivalent subjects

Search modes - Boolean/Phrase Interface - EBSCOhost Research Databases

Search Screen - Advanced Search

Database - CINAHL Plus 58 Edit S36

S35 "combined assessment unit*" Expanders - Apply equivalent subjects

Search modes - SmartText Searching Interface - EBSCOhost Research Databases

Search Screen - Advanced Search

Database - CINAHL Plus 22,561 Edit S35

S34 "combined assessment unit*" Expanders - Apply equivalent subjects

Search modes - Boolean/Phrase Interface - EBSCOhost Research Databases

Search Screen - Advanced Search

Database - CINAHL Plus 0 Edit S34

S33 "combined admissions unit*" Expanders - Apply equivalent subjects

Search modes - SmartText Searching Interface - EBSCOhost Research Databases

Search Screen - Advanced Search

Database - CINAHL Plus 4,546 Edit S33

S32 "combined admissions unit*" Expanders - Apply equivalent subjects

Search modes - Boolean/Phrase Interface - EBSCOhost Research Databases

Search Screen - Advanced Search

Database - CINAHL Plus 0 Edit S32

S31 "acute planning unit*" Expanders - Apply equivalent subjects

Search modes - SmartText Searching Interface - EBSCOhost Research Databases

Search Screen - Advanced Search

Database - CINAHL Plus 17,068 Edit S31

S30 "acute planning unit*" Expanders - Apply equivalent subjects

Search modes - Boolean/Phrase Interface - EBSCOhost Research Databases

Search Screen - Advanced Search

Database - CINAHL Plus 0 Edit S30

S29 "acute medical ward*" Expanders - Apply equivalent subjects

Search modes - Boolean/Phrase Interface - EBSCOhost Research Databases

Search Screen - Advanced Search

Database - CINAHL Plus 171 Edit S29

S28 "acute assessment unit*" Expanders - Apply equivalent subjects

Search modes - Boolean/Phrase Interface - EBSCOhost Research Databases

Search Screen - Advanced Search

Database - CINAHL Plus 25 Edit S28

S27 "medical assessment unit* OR AMU" Expanders - Apply equivalent subjects

Search modes - SmartText Searching Interface - EBSCOhost Research Databases

Search Screen - Advanced Search

Database - CINAHL Plus 54 Edit S27

S26 "medical assessment unit* OR AMU" Expanders - Apply equivalent subjects

Search modes - Boolean/Phrase Interface - EBSCOhost Research Databases

Search Screen - Advanced Search

Database - CINAHL Plus 0 Edit S26

S25 "acute admissions unit* OR AMU" Expanders - Apply equivalent subjects

Search modes - SmartText Searching Interface - EBSCOhost Research Databases

Search Screen - Advanced Search

Database - CINAHL Plus 46 Edit S25

S24 "acute admissions unit* OR AMU" Expanders - Apply equivalent subjects

Search modes - Boolean/Phrase Interface - EBSCOhost Research Databases

Search Screen - Advanced Search

Database - CINAHL Plus 0 Edit S24

S23 "acute medical unit* OR AMU" OR (MH "Observation Units") Expanders - Apply equivalent subjects

Search modes - Boolean/Phrase Interface - EBSCOhost Research Databases

Search Screen - Advanced Search

Database - CINAHL Plus 747 Edit S23

S22 "acute medicine care" Expanders - Apply equivalent subjects

Search modes - Boolean/Phrase Interface - EBSCOhost Research Databases

Search Screen - Advanced Search

Database - CINAHL Plus 2 Edit S22

S21 "acute medicine unit" Expanders - Apply equivalent subjects

Search modes - Boolean/Phrase Interface - EBSCOhost Research Databases

Search Screen - Advanced Search

Database - CINAHL Plus 12 Edit S21

S20 (MH "Emergency Care") Expanders - Apply equivalent subjects

Search modes - Boolean/Phrase Interface - EBSCOhost Research Databases

Search Screen - Advanced Search

Database - CINAHL Plus 24,647 Edit S20

S19 "*EMERGENCY SERVICE, HOSPITAL" Expanders - Apply equivalent subjects

Search modes - Boolean/Phrase Interface - EBSCOhost Research Databases

Search Screen - Advanced Search

Database - CINAHL Plus 31,270 Edit S19

S18 "accident AND emergency" Expanders - Apply equivalent subjects

Search modes - Boolean/Phrase Interface - EBSCOhost Research Databases

Search Screen - Advanced Search

Database - CINAHL Plus 2,467 Edit S18

S17 "emergency medicine''" Expanders - Apply equivalent subjects

Search modes - Boolean/Phrase Interface - EBSCOhost Research Databases

Search Screen - Advanced Search

Database - CINAHL Plus 18,734 Edit S17

S16 "emergency department*" Expanders - Apply equivalent subjects

Search modes - Boolean/Phrase Interface - EBSCOhost Research Databases

Search Screen - Advanced Search

Database - CINAHL Plus 69,430 Edit S16

S15 (MH "Emergency Medicine") Expanders - Apply equivalent subjects

Search modes - Boolean/Phrase Interface - EBSCOhost Research Databases

Search Screen - Advanced Search

Database - CINAHL Plus 12,580 Edit S15

S14 "EMERGENCY RESPONDERS OR HOSPITAL DEPARTMENTS" OR (MH "Emergency Service") OR (MH "Hospitals, Public") Expanders - Apply equivalent subjects

Search modes - Boolean/Phrase Interface - EBSCOhost Research Databases

Search Screen - Advanced Search

Database - CINAHL Plus 64,201 Edit S14

S13 S7 OR S8 OR S9 OR S10 OR S11 OR S12 Expanders - Apply equivalent subjects

Search modes - Boolean/Phrase Interface - EBSCOhost Research Databases

Search Screen - Advanced Search

Database - CINAHL Plus 327,610 Edit S13

S12 "pharmacy intervention" Expanders - Apply equivalent subjects

Search modes - Boolean/Phrase Interface - EBSCOhost Research Databases

Search Screen - Advanced Search

Database - CINAHL Plus 71 Edit S12

S11 "pre-registration pharmacist*" Expanders - Apply equivalent subjects

Search modes - Boolean/Phrase Interface - EBSCOhost Research Databases

Search Screen - Advanced Search

Database - CINAHL Plus 9 Edit S11

S10 (MH "Pharmacy Technicians") Expanders - Apply equivalent subjects

Search modes - Boolean/Phrase Interface - EBSCOhost Research Databases

Search Screen - Advanced Search

Database - CINAHL Plus 897 Edit S10

S9 (MH "Pharmacists") Expanders - Apply equivalent subjects

Search modes - Boolean/Phrase Interface - EBSCOhost Research Databases

Search Screen - Advanced Search

Database - CINAHL Plus 15,800 Edit S9

S8 "PHARMACY SERVICE, HOSPITAL" OR (MH "Pharmacy Service") Expanders - Apply equivalent subjects

Search modes - Boolean/Phrase Interface - EBSCOhost Research Databases

Search Screen - Advanced Search

Database - CINAHL Plus 7,307 Edit S8

S7 "pharmac*" Expanders - Apply equivalent subjects

Search modes - Boolean/Phrase Interface - EBSCOhost Research Databases

Search Screen - Advanced Search

Database - CINAHL Plus 327,610 Edit S7

S6 S1 OR S2 OR S3 OR S4 OR S5 Expanders - Apply equivalent subjects

Search modes - Boolean/Phrase Interface - EBSCOhost Research Databases

Search Screen - Advanced Search

Database - CINAHL Plus 2,789 Edit S6

S5 (MH "Medication History") Expanders - Apply equivalent subjects

Search modes - Boolean/Phrase Interface - EBSCOhost Research Databases

Search Screen - Advanced Search

Database - CINAHL Plus 324 Edit S5

S4 "drug history" Expanders - Apply equivalent subjects

Search modes - Boolean/Phrase Interface - EBSCOhost Research Databases

Search Screen - Advanced Search

Database - CINAHL Plus 286 Edit S4

S3 "medical reconciliation" Expanders - Apply equivalent subjects

Search modes - Boolean/Phrase Interface - EBSCOhost Research Databases

Search Screen - Advanced Search

Database - CINAHL Plus 13 Edit S3

S2 "medication reconciliation" Expanders - Apply equivalent subjects

Search modes - Boolean/Phrase Interface - EBSCOhost Research Databases

Search Screen - Advanced Search

Database - CINAHL Plus 2,244 Edit S2

S1 (MH "Medication Reconciliation") Expanders - Apply equivalent subjects

Search modes - Boolean/Phrase Interface - EBSCOhost Research Databases

Search Screen - Advanced Search

Database - CINAHL Plus 1,790 Edit S1

# Healthcare Management of Information Consortium (HMIC) – via UoB 15/11/2020

1. exp medicines management/ 434
2. ''medication reconciliation''.ti,ab. 21
3. ''medical reconciliation''.ti,ab. 0
4. ''drug history''.ti,ab. 22
5. ''medication history''.ti,ab. 28
6. 1 or 2 or 3 or 4 or 5 490
7. ''pharmac*.ti,ab. 9238
8. exp Pharmacists/ 2218
9. exp Pharmacy technicians/ 72
10. ''pre-registration pharmacist*''.ti,ab. 3
11. ''pharmacy intervention''.ti,ab. 4
12. 7 or 8 or 9 or 10 or 11 9483
13. exp Patient emergency admissions/ 953
14. exp Hospital departments/ 5724
15. ''emergency department*''.ti,ab. 2085
16. ''emergency medicine*''.ti,ab. 165
17. (''accident and emergency'').ti,ab. 1810
18. ''emergency care''.ti,ab. 637
19. ''acute medicine unit''.ti,ab. 6
20. ''acute medicine care''.ti,ab. 0
21. (''acute medical unit*'' or AMU).ti,ab. 60
22. (''acute admissions unit*'' or AMU).ti,ab. 30
23. (''medical assessment unit*'' or AMU).ti,ab. 60
24. ''acute assessment unit*''.ti,ab. 5
25. ''acute medical ward*''.ti,ab. 47
26. ''acute planning unit*''.ti,ab. 0
27. ''combined admissions unit*''.ti,ab. 0
28. ''combined assessment unit*''.ti,ab. 0
29. ''medical admissions unit*''.ti,ab. 30
30. ''rapid assessment medical unit*''.ti,ab. 31
31. 13 or 14 or 15 or 16 or 17 or 18 or 19 or 20 or 21 or 22 or 23 or 24 or 25 or 26 or 27 or 28 or 29 or 30 9445
32. 6 and 12 and 31 12

# Clinical Trails.Gov 15/11/2020

Terms Search Results* Entire Database**

Synonyms

medication reconciliation 137 studies 137 studies

reconciliation 140 studies 167 studies

medication 140 studies 207,998 studies

Drug 88 studies 172,841 studies

Medicine 54 studies 53,063 studies

Pharmaceutical 31 studies 21,599 studies

medicinal products 1 studies 2,085 studies

Pharmaceutic Preparations -- 1 studies

# International Clinical Trials Registry Platform (ICTRP) 12/01/2022

Medication reconciliation = 0

Medication = 0

Reconciliation = 0

Pharmac*= 0

Pharmacy = 0

Emergency Medicine = 0

# CENTRAL and CDSR using Medline search (via Cochrane) 29/11/2020

Search help View saved searches Save this search

Print

View fewer lines

1. MeSH 81
2. S Limits 347
3. S Limits 185
4. S Limits 22117
5. S Limits 6611
6. Limits 25731
7. S Limits 223116
8. MeSH 139
9. MeSH 580
10. MeSH 18
11. S Limits 2
12. S Limits 2459
13. Limits 223116
14. MeSH 2383
15. MeSH 3550
16. MeSH 269
17. MeSH 301
18. S Limits 11173
19. S Limits 3014
20. S Limits 857
21. S Limits 11416
22. S Limits 425
23. S Limits 1547
24. S Limits 3085
25. S Limits 423
26. S Limits 5787
27. S Limits 3453
28. S Limits 937
29. S Limits 213
30. S Limits 89
31. S Limits 1911
32. S Limits 515
33. S Limits 167
34. Limits 31666
35. Limits 627

# Web of Science 29/11/2020 forward citation completed 25/05/2021

You searched for: TOPIC: (''medication reconciliation'') AND TOPIC: (''pharmac*'') AND TOPIC: (''hospital'')

Timespan: All years. Indexes: SCI-EXPANDED, SSCI, A&HCI, CPCI-S, CPCI-SSH, BKCI-S, BKCI-SSH, ESCI, CCR-EXPANDED, IC.
